# Natural Compounds and Derivatives as Ser/Thr Protein Kinase Modulators and Inhibitors

**DOI:** 10.3390/ph12010004

**Published:** 2019-01-01

**Authors:** Barbara Guerra, Olaf-Georg Issinger

**Affiliations:** Department of Biochemistry and Molecular Biology, University of Southern Denmark, 5230 Odense M, Denmark

**Keywords:** protein kinase, cancer, phosphorylation, natural compounds, flavonoids, kinase inhibition, cellular signalling

## Abstract

The need for new drugs is compelling, irrespective of the disease. Focusing on medical problems in the Western countries, heart disease and cancer are at the moment predominant illnesses. Owing to the fact that ~90% of all 21,000 cellular proteins in humans are regulated by phosphorylation/dephosphorylation it is not surprising that the enzymes catalysing these reactions (i.e., protein kinases and phosphatases, respectively) have attracted considerable attention in the recent past. Protein kinases are major team players in cell signalling. In tumours, these enzymes are found to be mutated disturbing the proper function of signalling pathways and leading to uncontrolled cellular growth and sustained malignant behaviour. Hence, the search for small-molecule inhibitors targeting the altered protein kinase molecules in tumour cells has become a major research focus in the academia and pharmaceutical companies.

## 1. Introduction

The search for anticancer agents from plant sources goes back to the 1950s with the discovery and development of the vinca alkaloids—vinblastine and vincristine—and the isolation of the cytotoxic podophyllotoxins [1]. From the 1940s to the end of 2014, one-hundred-and-seventy-five small molecule inhibitors were approved for treatment of cancer. One-hundred-and-thirty-one are non-synthetic, with 85 being either natural products or directly derived there from [2]. As early as 1982 it was shown that quercetin and related polyphenols selectively inhibited protein kinase CK2 in contrast to PKA that was not affected [3]. Graziani et al. [4] showed that quercetin inhibited the phosphotransferase activity of the Rous sarcoma virus gene product, pp60src. The bacterial alkaloid staurosporine was shown to inhibit PKC in the nanomolar range [5]. Thirty-five years ago the inhibitory efficacy of bioflavonoids and other natural products on certain protein kinases was established, albeit as an interesting tool in the biochemical characterization of the different types of protein kinases in whole cell extracts. 

The importance of natural compounds and their effect on protein kinases with respect to inhibition of tumour growth came, understandably, to the attention of the medical community much later. Currently, the Food and Drug Administration (FDA) has approved over 40 drugs including orally effective compounds directed against protein kinases, that target a limited number of these enzymes (http://www.brimr.org/PKI/PKIs.htm, [6]). Approximately 150 kinase-targeted drugs are currently in clinical phase trials, and many kinase-specific inhibitors are in the preclinical stage of drug development. It is of interest to note that kinase inhibitors have a wide range of possible targets. Some approved antitumour-directed kinase inhibitors have a very large number of “targets” in the human kinome. Hence, it is not surprising that kinase inhibitors may also affect other signalling molecules relevant in other diseases.

## 2. Protein Kinases

Protein kinases represent a broad group of evolutionary- and structurally-related enzymes. They preferably catalyse the phosphorylation of serine/threonine and tyrosine residues in their substrates. These hydroxy/phenolic amino acids are embedded in a short recognition sequence of amino acids typical for protein kinases. With a group size of 518 individual members, the “kinome”, that is the sum of all kinase-coding genes, constitutes ~2% of the human genome [7]. Since the isolation of the first serine/threonine-specific kinase (STK) in the muscle in 1959, it took another 20 years until tyrosine protein kinases were discovered. Although all these enzymes differ in size, substrate specificity, mechanism of activation, subunit composition and subcellular localization, they all share a highly conserved homologous catalytic core [8]. Most protein kinases target both serine and threonine (serine/threonine kinases, STKs) residues, others act on tyrosine (tyrosine kinases, TKs) and a number phosphorylate all three types of amino acids (i.e., dual-specificity kinases, DSKs) [9]. The kinome is organised in the following subfamilies of protein kinases, i.e., AGC, CaMK, CK1, CMGC, STE, TK and TKL [10]. The first estimates were that ~30% of all proteins become phosphorylated, however, recent studies suggest that 90% of the 21,000 proteins encoded by the human genome are subjected to this type of post-translational modifications [11].

Phosphatases are enzymes reversing the phosphorylation state of proteins. Currently there are approximately 226 known protein phosphatases [12]. The process of phosphorylation and dephosphorylation can be extremely complex, since a single kinase or phosphatase may simultaneously have multiple substrates and may function in various cell-signalling pathways. The concept of reversible protein phosphorylation developed by Fischer and Krebs with the elucidation of the hormonal control of glycogenolysis [13] is the basis for the understanding of how cellular functions are controlled, a finding that brought E.G Krebs and E. Fischer the Nobel Prize in Medicine in 1992.

## 3. Protein Kinases in Cellular Signalling

The signalling pathways regulated by protein kinases contribute to the onset and progression of almost all types of cancer. Consequently, research of the signalling pathways mediated by protein kinases and the possibility of blocking them with targeted treatment does have major clinical/therapeutic utility especially since many of these proteins act as oncogenes. The signalling networks operating in cancer cells can also contribute to innate or acquired resistance to treatment, since they are able to create the most common or rare oncogenic mutations different from tumour to tumour (i.e., the so-called polygenic tumour biology, [14,15]).

**Figure 1:**
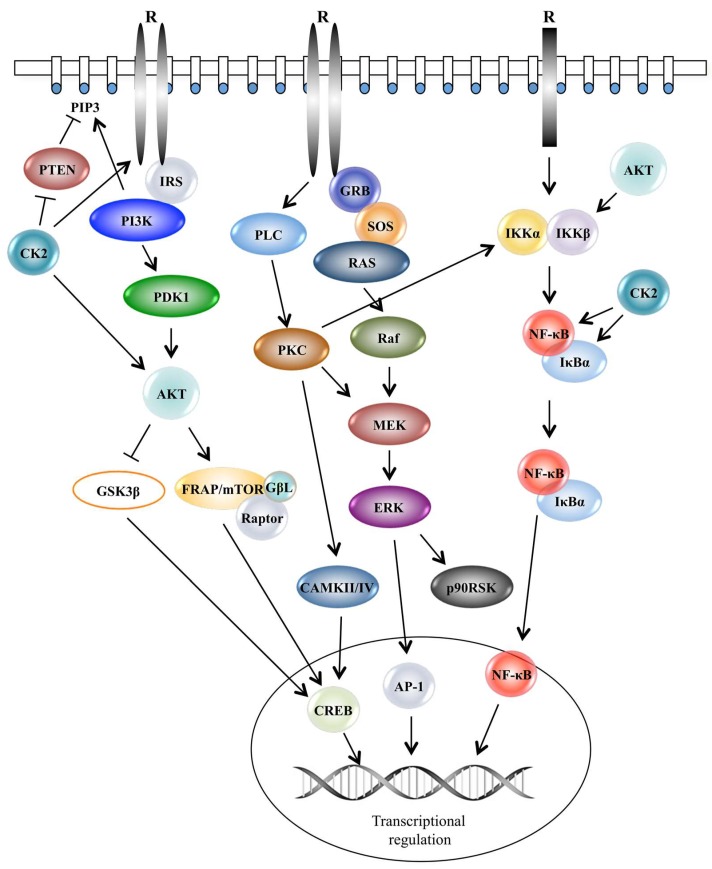
Schematic representation of intracellular signal transduction pathways supporting cell proliferation and survival. Activation of the PI3K/AKT/mTOR, RAS/Raf/MEK/ERK and IKK/NF-κB pathways occurs following a stimulus represented by a ligand, which binds a receptor tyrosine kinase located on the plasma membrane. These pathways control gene expression in a number of ways comprising phosphorylation of transcription factors and co-factors and modification of protein-binding DNA. R: Receptor; PTEN: phosphatase and tensin homolog; IRS: insulin receptor substrate; PI3K: phosphatidylinositol-3-kinase; PDK1: phosphoinositide-dependent-kinase-1; CK2: protein kinase CK2; AKT: protein kinase B; GSK3β: glycogen synthase kinase 3; FRAP/mTOR: 12-rapamycin-associated protein 1/mammalian target of rapamycin; GβL: G protein beta subunit-like; GRB: growth factor receptor-bound protein 2; SOS: son of sevenless; Raf: rapidly accelerated fibrosarcoma; PKC: protein kinase C; MEK: mitogen-activated protein kinase; ERK: extracellular signal-regulated kinase; CAMKII/IV: calcium-calmodulin kinase II/IV; p90RSK: p90 ribosomal s6 kinase; IKKα: IκB kinase α-subunit; IKKβ: IκB kinase β-subunit; NF-κB: nuclear factor-kappa B; IκBα: inhibitor of kappa B; AP-1: activator protein-1; CREB: cAMP response element-binding protein.

Most cancer-relevant natural products interact with molecules involved in key intracellular signalling pathways like PI3K/AKT/mTOR/GSK3β, Ras/Raf/MEK/MAPK/ERK1/2, PL-Cγ/PKC/CaMKII/IV or JNK just to mention the classical signalling cascades. They all have in common that they influence key molecules in the nucleus mostly proteins involved in the regulation of gene transcription. Yet, these transcription factors are also subject to interaction with small natural compounds. Most molecules in the signalling pathways shown in Figure 1 belong to the STK group. Their activation, however, occurs following stimulation of tyrosine kinase receptors located in the cell plasma membrane and become active by their binding of external ligands on the extracellular domain of the receptor molecule (Figure 1).

## 4. Role of Protein Kinases in Cancer

As already mentioned, protein kinases recognise specific short amino acid sequences in their substrates [16]. Some protein substrates, like the well-characterised tumour suppressor p53 protein is targeted by more than 30 different protein kinases and at more than 20 sites. Some sites, e.g., Ser15, are phosphorylated by more than eight different protein kinases. Needless to say that the elucidation of which protein kinase is responsible for the phosphorylation at a particular site at a certain time point is a challenging task [17].

**Figure 2:**
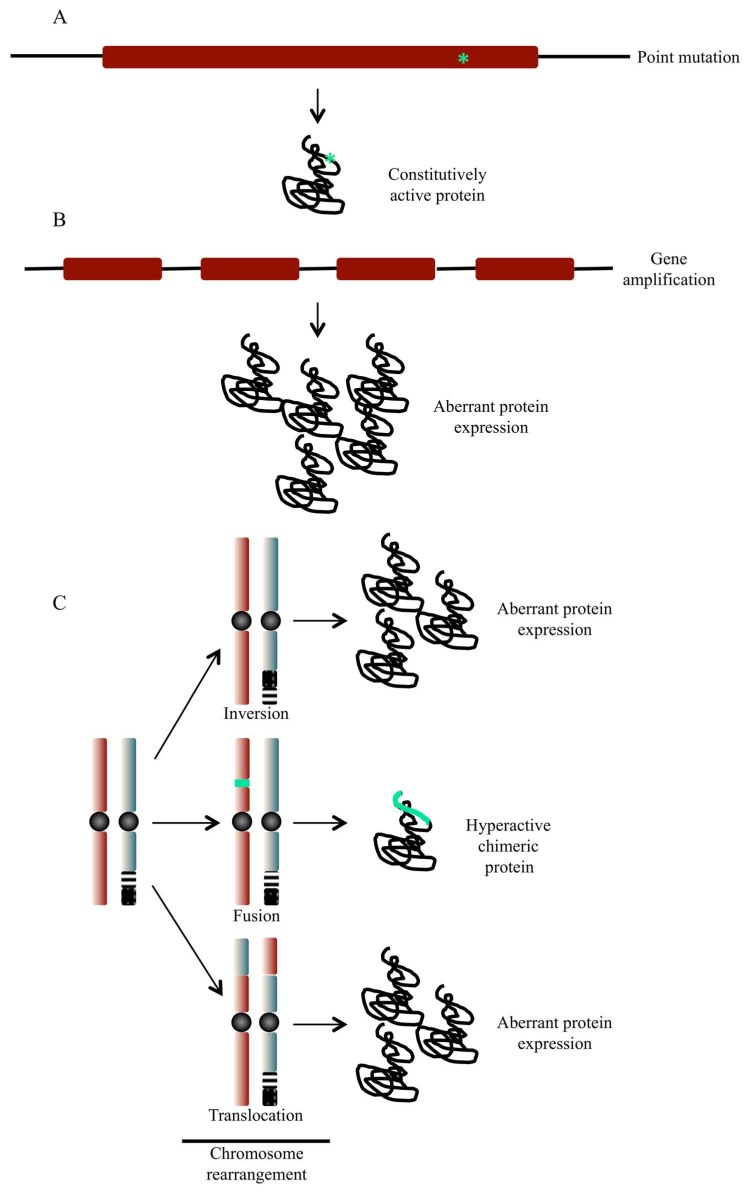
Possible alterations of protein kinase-coding genes and their outcome. (**A**) Point mutations are capable of activating a proto-oncogene product resulting in the expression of a constitutively active protein kinase. (**B**) In addition to nucleotide substitution, gene amplification that results from amplification of chromosomal fragments can result in aberrant expression of genes coding for protein kinases. This can result in uncontrolled activation of signalling pathways controlled by the overexpressed protein kinases. (**C**) Alteration in the structure of chromosomes can take several forms including translocation, inversion, deletion and insertion of genetic material. Chromosome abnormalities can result in the expression of higher levels of a protein, chimeric hyperactive proteins or loss of tumour suppressor gene products.

The interesting aspect of these considerations is that many proteins involved in signal transduction are targets for protein kinase modification and, most importantly, their function and/or activity can be modified rapidly, without the need of genetic alterations. The process of phosphorylation/dephosphorylation is a very efficient and rapid way of altering the activity of signalling molecules and one can imagine that mutations of either protein kinases or substrate molecules may lead to aberrations in signalling pathways important for preserving normal cellular homeostasis. The initial causes of cancer are multiple, including prolonged hormone stimulation (e.g., oestrogens in breast cancers and androgens in prostate cancers), viruses, chemical carcinogens, radiation, persistent inflammation, acquired or inborn genetic events and aneuploidy. Cancer is regarded as a genetic disease involving oncogenes activated by amplification or by point mutations. Chromosomal aberrations, which result in alterations in oncogenes and tumour suppressor genes in somatic cells, are another possible cause leading to cancer induction in humans and experimental animals (Figure 2). The abnormal oncogenic activation of protein kinases derives from multiple types of genetic changes. These alterations result in increased specific activity of the kinases themselves, their overexpression, or the loss of negative regulation. Most frequently, cancer cells harbour somatic point mutations at structurally conserved residues, or mutation in hotspot regions, which constitutively upregulate the kinase activity [18]. In addition, genomic instability—a hallmark of cancer cell [19,20]—can also result in elevated kinase activity enhancing intracellular signalling through a number of distinct mechanisms. Defects in the surveillance pathways that maintain genomic integrity can produce amplifications of large chromosomal regions or complex chromosomal rearrangements, which in turn result in a defect expression of a protein kinase or the expression of a constitutively activated chimeric form.

The abnormal oncogenic activation of protein kinases can also derive from multiple epigenetic [21,22], transcriptional or post-transcriptional changes as a result from alternative splicing regulation and miRNA expression (Figure 2, [9]).

The original idea of a ‘magic bullet’ to cure cancer that is the design of drugs that directly target the intended cell component has been largely abandoned. Mounting evidence suggests that signalling pathways that become dysfunctional by altered molecules should be tackled by combination therapy including multiple kinase inhibitors [23]. There are various mechanisms by which kinase inhibitors bind to their target kinases broadly classified into kinase inhibitors that bind covalently or noncovalently to or around the ATP binding site. Primarily, protein kinases interact with ATP through a cleft located between the N- and C-terminal lobes of the kinase domain. However, specific tumour genetics, tumour microenvironment, drug resistance and pharmacogenomics determine how effective a compound will be in the treatment of a specific cancer.

The signalling pathways in cells in a given organism are unique and detailed knowledge on their components can be used to find drugs to influence them based on the diseases that should be tackled. In other words a drug that was designed to treat, for instance, cancer also can have a positive effect on diabetes. These observations led to a more holistic insight concerning treatment of various major diseases. The concept of this review is to give place for a complementary therapeutic research approach, based on one hand on western medicine and on the other hand on knowledge from traditional Chinese medicine (TCM).

## 5. Natural Bioactive Compounds as Kinase Inhibitors

There is evidence that the medicinal use of natural products derived from sources such as plants, animals or microorganisms dates back to the Neanderthal period [24,25]. Owing to the diverse biological activities of the isolated natural products all major civilizations have accumulated knowledge and experience in their application. During the 19th century modern Western chemistry succeeded in the identification of the active ingredients from natural sources leading to the isolation, for instance, of morphine, salicin, emetine strychnine, colchicine, caffeine, nicotine, cocaine, etc. Moreover, the elucidation of their molecular structure allowed the synthesis of the desired compounds instead of isolating them from natural sources [26]. This procedure also had the advantage to be cheaper and, moreover, allowed to use the active ingredient of a medicinal plant and not the crude plant extract. Newman [27] estimates that ~60% of the drugs that are nowadays available—including household names such as artemisinin, camptothecin, lovastatin, maytansine, paclitaxel, penicillin, reserpine and silibinin—were either directly or indirectly derived from natural products.

Plants provide an extensive reservoir of natural products. The significance of natural products in health care is supported by a report that 80% of the global population still relies on plant-derived medicines to address their health care needs.

In recent years, there has been a major paradigm shift in discovery and screening of natural products as potential kinase inhibitors. Protein kinase inhibitors belong chemically to a variety of plant compounds such as flavonoids, alkaloids, sesquiterpenes, diterpenoids and polyphenolics, which represent a large and diverse group of naturally occurring compounds found in a variety of fruits, vegetables and medicinal plants with various anticancer properties.

In contrast to TCM, the Western approach is more specific and targeted, although one has to admit that the “magic bullet” that has never been found, and probably will not be found, simply because cancer is a complex genetic disease.

Danggui Longhui Wan (DLW) is a typical traditional Chinese medicinal prescription for the treatment of chronic myeloid leukaemia (CML) [28,29]. It is made up of 11 herbal medicines. The search for the active compound revealed that only indirubin was effective against CML. The remaining 10 compounds were all inactive. Indirubin was identified as the active ingredient [28]. Indirubin and its analogues are potent inhibitors of cyclin-dependent kinases (CDKs) involved in cell division [30]. Since tumour cells heavily depend on cell division, the inhibition of CDKs will block progression of cell division and so tumour growth. However, indirubin worked well in cell-free tests but proved difficult to be absorbed in the intestinal tract. We will let this stand as one example, where the analysis of TCM can be rewarding to identify the active compound(s). Once identified, they can be further “improved” by chemical modifications. However, as in the case of DLW, one should not dismiss the other ten compounds as useless. Quite the contrary, especially, since the mixture has worked successfully in treating CML in the last two thousand or more years. One can imagine that there are compounds present in the mixture, which might increase the solubility of the active components that by themselves are insoluble. Therefore, TCM that is based on herb mixtures could be a role model for finding an active ingredient and together with complementary compounds it would finally make it a magic bullet.

## 6. Classification of Flavonoids and Other Polyphenolic Compounds

Polyphenols have been credited with their antioxidant properties targeting protein kinases in general through interaction with the ATP-binding site. Lolli et al. [31] have crystallised different flavonoids together with protein kinase CK2α. The authors showed that apigenin and luteolin, when modelled into the CK1 delta isoform were fitting well. This fit could apply for many other kinases. Baier et al. [32,33] modelled two CK2 isoforms with different flavonoid compounds. Their results support the notion that the tested flavonoids are purely ATP competitive. Especially, apigenin, which is a relative simple flavonoid, could be a role model for other flavonoids. Thirty-four PDB entries correspond to more than 2000 kinase and more than 150 flavonoid entries (one should keep in mind that in these PDB entries the host protein is not necessarily a protein kinase).

In summary we can say that protein kinases can be inhibited by flavonoids through ATP competitive inhibition. This is because of the molecular basic structure of flavonoids that fits well into the ATP binding pocket and the neighbouring hinge region, which provides H-bonds for interactions, yet, this interaction may not apply to all flavonoids.

Polyphenols comprise a wide variety of molecules that have a polyphenol structure, i.e., several hydroxyl groups on aromatic rings, but also molecules with one phenol ring. Polyphenols are subdivided into several major subclasses: phenolic acids, stilbenes, tannins, diferuloylmethanes and flavonoids. There are six major subgroups of flavonoids. They are the most abundant antioxidants in our diet and are common constituents of foods of plant origin and are wide spread constituents of fruits, vegetables, cereal, olive, dry legumes, chocolate and beverages such as tea, coffee and wine [34]. Inhibition of cell growth, antiapoptotic properties, inhibition of tumour cell invasion and inhibition of protein kinase activities are part of the antitumour activities of flavonoids. It is interesting to note that bioflavonoids are by far the largest natural compound group inhibiting protein kinases. In 1994 more than 500 types of flavonoids were reported [35]. Now, 24 years later, the number has grown to ~9000 [36]. Flavones and flavonols contain the largest number of compounds within the group of bioflavonoids. We are presenting here only a selection of the most described members of the flavonoid family and other polyphenol compounds with respect to their effect on protein kinases and cancer.

*Flavones*

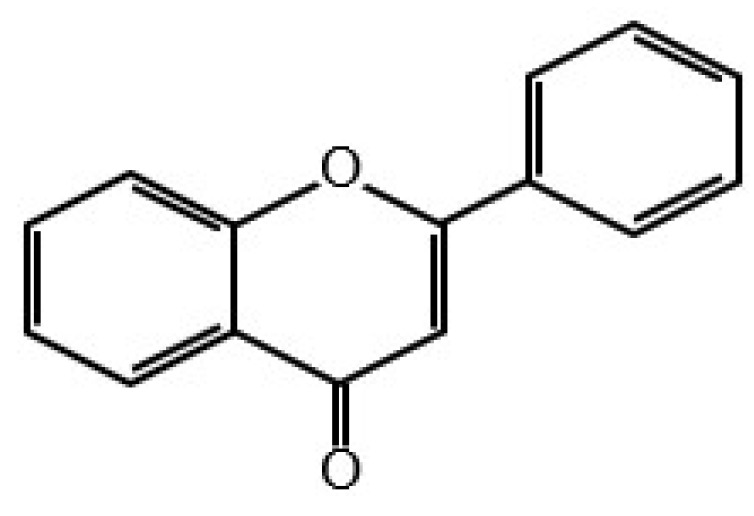



Apigenin is a flavone present in vegetables such as parsley, celery and chamomile [37]. It acts as an effective anticancer natural product on TRAIL, WNT/β-catenin signalling, the JAK-STAT pathway and microRNAs. Moreover, it was shown that it targets the PI3K/AKT/mTOR signalling axis for cancer prevention [38].

Apigenin also has been shown to inhibit prostate cancer progression in TRAMP mice by targeting the PI3K/AKT/FoxO pathway [39], block IKKalpha activation and suppress prostate cancer progression [40].

Luteolin is a type of flavonoid that inhibits proliferation involving MAPK and mTOR signalling pathways in various tumour cell lines [41].

Baicalein is another flavone targeting prosurvival signalling pathways. It is one of the major active constituents of *Scutellariae radix*. In TCM it is known as Huang Qin and used to treat various diseases. The antitumour effects of baicalein are mainly due to its ability to inhibit cyclin-containing complexes involved in cell cycle regulation, to scavenge oxidative radicals and to attenuate mitogen activated protein kinase (MAPK), AKT and mTOR activity [42].

Most of the flavones listed in Table 1 share similar targets as described for apigenin.

*Flavonols*

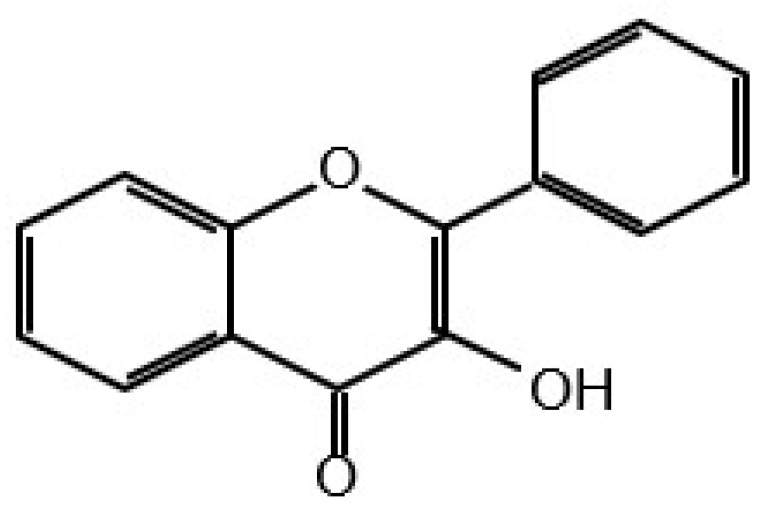



Quercetin has been found in apples, berries, red onions, cherries, broccoli, coriander, etc. [43]. Beside TKs, it has been shown to inhibit the serine/threonine kinases, AKT, mTOR, MAPK, ERK1/2, JNK, etc. This indicates that this compound inhibits members of major pro-survival signalling pathways as shown in Figure 1. A linked search for the number of citations found for quercetin, protein kinases and cancer amounted to 344.

Kaempferol. Common foods that contain kaempferol include apples, grapes, tomatoes, green tea, potatoes, onions and broccoli, just to name a few fruits and vegetables. Kaempferol has been shown to inhibit angiogenesis by targeting VEGF receptor-2 and down-regulating the PI3K/AKT, MEK and ERK pathways [44]. Moreover, kaempferol inhibits invasion and migration of renal cancer cells through the downregulation of AKT and FAK pathways [45] and it increases apoptosis in human cervical cancer HeLa cells via the PI3K/AKT and hTERT pathways [46].

Fisetin has been found in strawberries, apples, persimmons, onions and cucumbers. It has shown anticancer activity on mammary carcinoma cells via regulation of the PI3K/AKT/mTOR pathway [47]. It inhibits laryngeal carcinoma through regulation of AKT/NF-κB/mTOR and ERK1/2 signalling cascades [48] Moreover, it has been shown that fisetin induces apoptosis in human non-small cell lung cancer through inhibition of the MAPK signalling cascade [49].

*Flavanones*

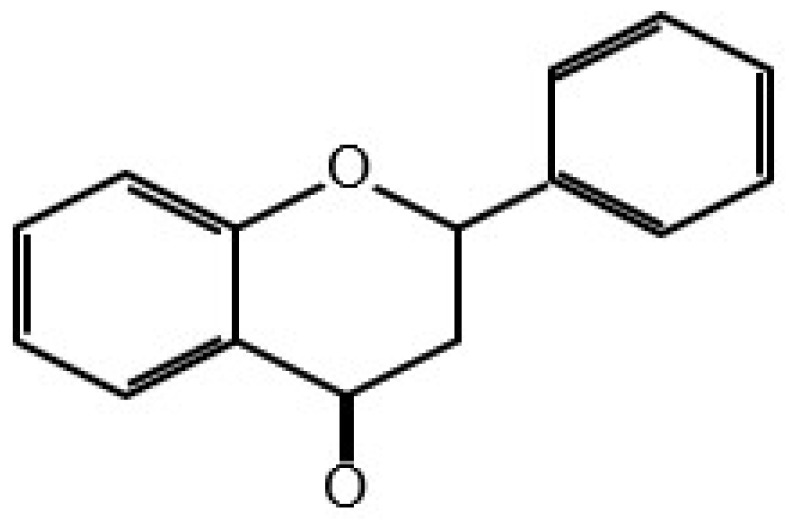



Naringenin. Almost exclusively found in citrus fruits, especially in grapefruit. Cytotoxicity has been induced by naringenin in cancer cells from breast, stomach, liver, cervix, pancreas and colon tissues, along with leukaemia cells. Naringenin has been shown to induce cell death in prostate cancer cells via PI3K/AKT and MAPK signalling pathways [50]. Moreover, it was shown that this compound inhibits cell proliferation, migration and invasion in gastric cancer cells by downregulation of the AKT pathway [51].

*Flavanonols*

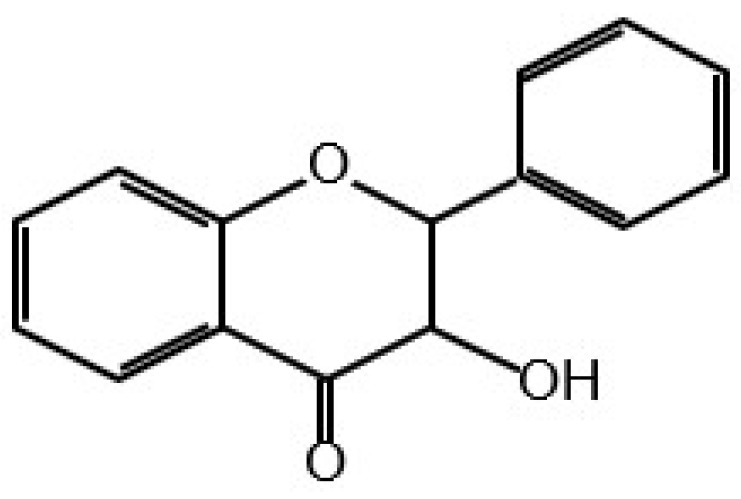



Silibinin. Silymarin, which is extracted from the seeds and fruits of the milk thistle *Silybum marianum* is a mixture of three structural isomers, silibinin, silidianin and silichristin. Silibinin induces cell cycle arrest in G1 phase, apoptosis and JNK/SAPK upregulation in SW1990 human pancreatic cancer cells [52]. Moreover, it was shown that combined treatment with sorafenib and silibinin synergistically targets both hepatocellular carcinoma cells and cancer stem cells by enhanced inhibition of the phosphorylation of STAT3/ERK/AKT [53].

Taxifolin. This compound can be found in conifers like the siberian larch, *Larix sibirica*, in Russia, in *Pinus roxburghii*, in *Cedrus deodara* and in the Chinese yew, *Taxus chinensis var. mairei*. It is also found in the silymarin extract from the milk thistle seeds. Taxifolin inhibits growth, migration and invasion of human osteosarcoma cells by reducing expression levels of AKT [54]. Taxifolin suppresses UV-induced skin carcinogenesis by targeting EGFR and PI3K [55].

*Isoflavones*

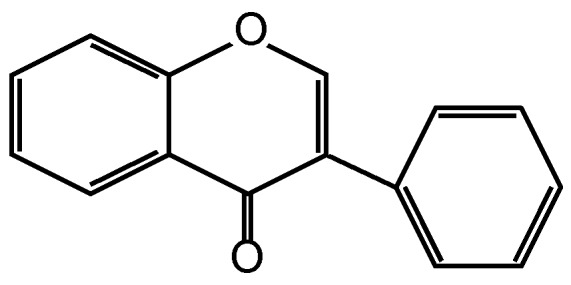



Genistein and daidzein are found in a number of plants including lupine, fava beans and soybeans. Genistein reduces the activation of AKT and EGFR, and the production of IL6 in cholangiocarcinoma cells involving oestrogen and oestrogen receptors [56]. Inactivation of AKT, ERK and NF-κB by genistein reduces ovarian carcinoma oncogenicity [57]. Moreover, it was shown that genistein inhibits the growth and regulates the migration and invasion abilities of melanoma cells via the FAK/paxillin and MAPK pathways [58]. Taking everything into consideration, genistein acts as a chemotherapeutic agent against different types of cancer, mainly by altering apoptosis, the cell cycle and angiogenesis and inhibiting metastasis. It appears so that the therapeutic effects of genistein very often result from targeting extracellular signal-regulated kinase 1/2 (ERK1/2), mitogen-activated protein kinase (MAPK), inhibitor of NF-κB (IκB) and PI3K/AKT signalling pathways.

*Flavan-3-ols/Catechins*

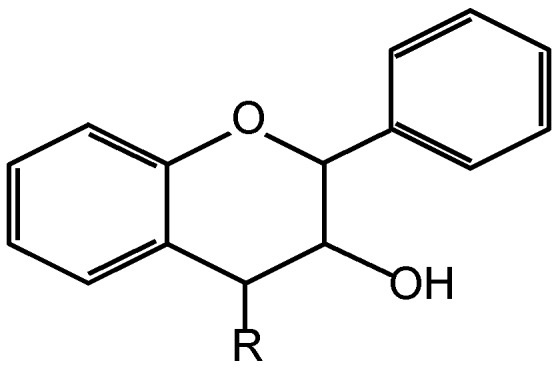



Epigallocatechin-3-gallate (EGCG) is found in high content in the dried leaves of green tea, white tea and in smaller quantities, black tea. EGCG downregulates doxorubicin-induced overexpression of P-glycoprotein through the coordinate inhibition of PI3K/AKT and MEK/ERK signalling pathways [59]. Moreover, it was shown that the compound can promote apoptosis in human breast cancer T47D cells through downregulation of the PI3K/AKT signalling cascade [60,61].

*Anthocyanins*

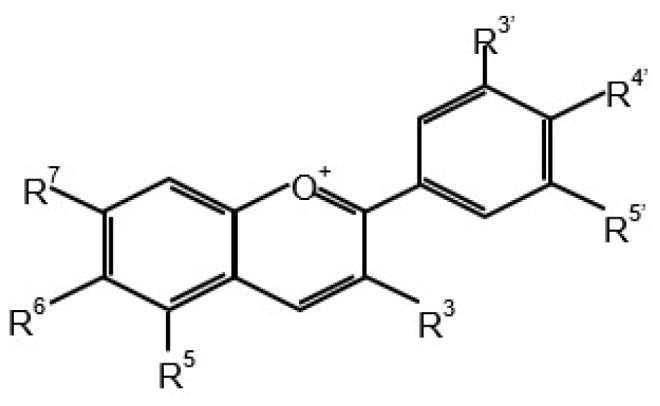



Delphinidin is an anthocyanidin, a primary plant pigment. It is also an antioxidant and can be found in cranberries and concord grapes as well as pomegranates and bilberries. The compound induces apoptosis and inhibits epithelial-to-mesenchymal transition via the ERK/p38 MAPK-signalling pathway in human osteosarcoma cell lines [62]. Delphinidin also influences the proliferation of ovarian cancer cells via PI3K/AKT and ERK1/2 MAPK signalling axis [63] Moreover, it was shown that this compound induces autophagy in HER2+ breast cancer cells via inhibition of AKT/mTOR pathway [64]. 

*Sesquiterpenes*

Parthenolide 

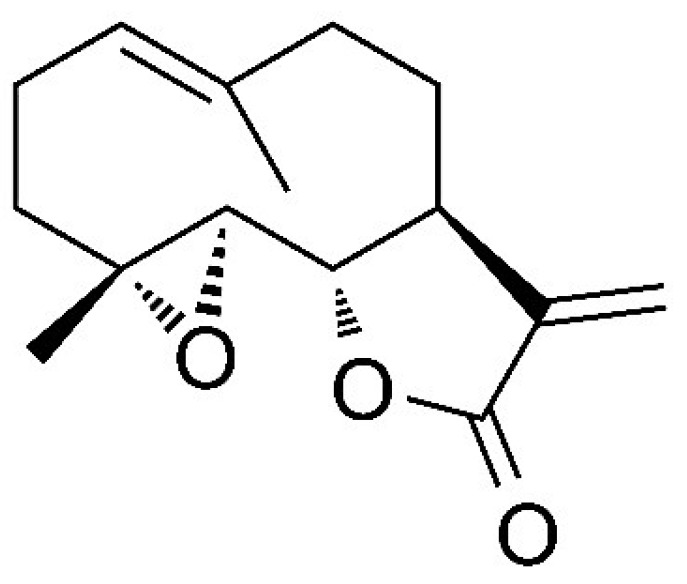



Parthenolide is a sesquiterpene lactone of the germacranolide class, which occurs naturally in the plant feverfew (*Tanacetum parthenium*), after which it is named. It is found in highest concentration in the flowers and fruit. Parthenolide suppresses non-small cell lung cancer GLC-82 cell growth via B-Raf/MAPK/ERK pathway [65]. Moreover, it induces apoptosis and autophagy through the suppression of PI3K/AKT signalling cascade in cervical cancer [66].

*Diterpenes*

Oridonin

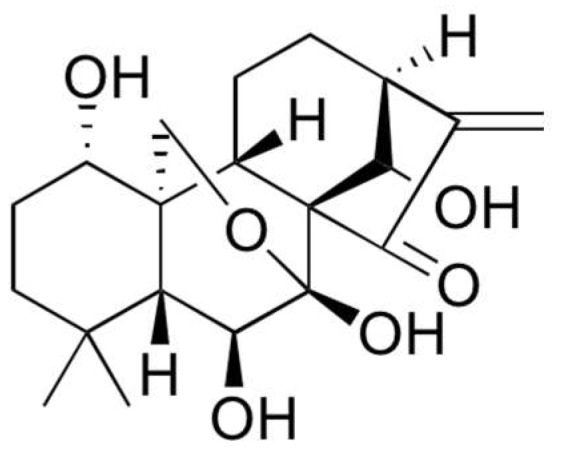



Oridonin is an organic heteropentacyclic compound isolated from the leaves of the medicinal herb *Rabdosia rubescens*. It has a role as an antineoplastic agent, an angiogenesis inhibitor, an apoptosis inducer, an anti-asthmatic agent, a plant metabolite and an antibacterial agent. Oridonin inhibits oral cancer growth and PI3K/Akt signalling pathway [67]. Moreover, the compound has shown enhanced anticancer efficacy by inhibiting PI3K/AKT and Ras/Raf/MEK/ERK pathways [68]. Oridonin upregulates PTEN through activating p38 MAPK and inhibits proliferation in human colon cancer cells [69].

*Neolignan biphenol*

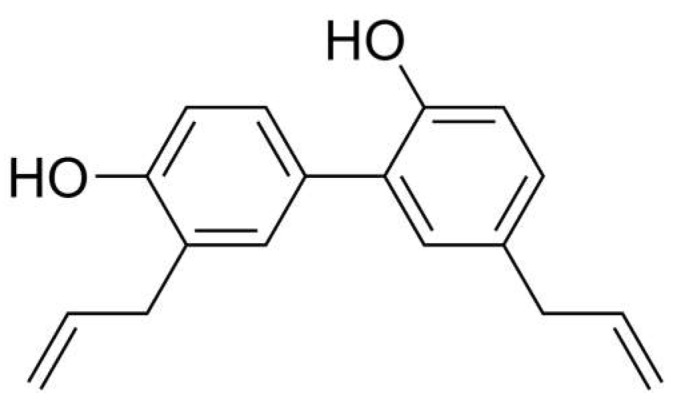



Honokiol is a lignan isolated from the bark, seed cones and leaves of trees belonging to the genus Magnolia. It suppresses proliferation and induces apoptosis via regulation of the PTEN/PI3K/AKT pathway in human osteosarcoma cells [70]. It has also been shown to induce autophagy and apoptosis in osteosarcoma cells through PI3K/AKT/mTOR signalling pathway [71]. Moreover, it has been reported en effect on proliferation, migration and invasion in nasopharyngeal carcinoma cells [72]. Taken everything into consideration, honokiol has shown proapoptotic effects in melanoma, sarcoma, myeloma, leukaemia, bladder, lung, prostate, oral squamous cell carcinomas, in glioblastoma multiforme cells and colon cancer cell lines. Honokiol inhibits phosphorylation of Akt, p44/42 mitogen-activated protein kinase (MAPK) and Src kinase. It also acts on the PI3K/AKT/mTOR pathway in tumour cells while maintaining pathway activity in T cells.

*Coumestans*

Wedelolactone (7-methoxy-5,11,12-trihydroxy-coumestan)

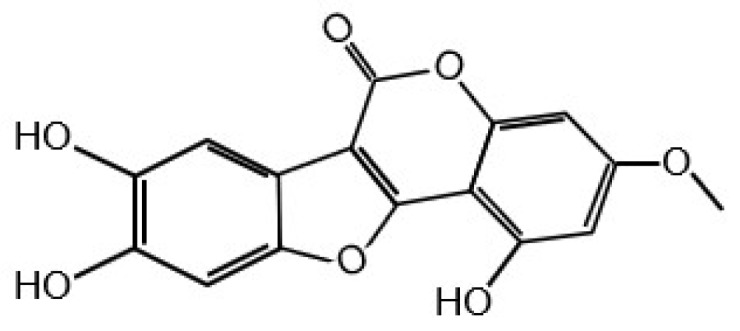



It is a plant-derived natural product synthesized mainly by members belonging to the *Asteraceae* family. Wedelolactone inhibits breast cancer-induced osteoclastogenesis by inhibiting AKT/mTOR signalling [73]. It also has been shown to induce caspase-dependent apoptosis in prostate cancer cells via downregulation of PKCε and without inhibition of AKT kinase. 

*Alkaloids*

Curcumin from turmeric 

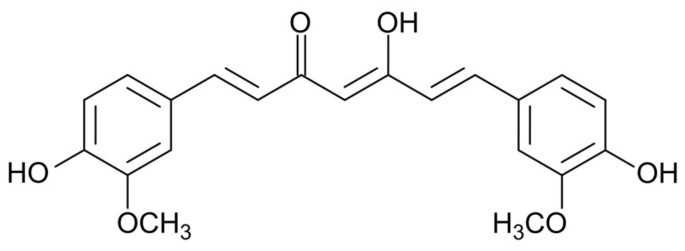



Curcumin (diferuloylmethane) the major ingredient of the popular Indian spice turmeric, *Curcuma longa L.*, is a member of the ginger family.

Curcumin, is a polyphenol with molecular targets for cancer control [74] and has a multifaceted role in cancer prevention and treatment [75]. The following reviews focus on the role of curcumin in cancer and treatment with respect to the PI3K/AKT pathway [76] and its antiangiogenic activity in cancer therapy [77], respectively. 

## 7. Protein Kinase Inhibition by Natural Compounds. Is This an Option?

Polyketides are a heterogeneous group of secondary metabolites. We have focused in this review on the largest group of these compounds, which are the flavonoids with more than 9000 known compounds today. There are other polyketides such as stilbenes and curcuminoids which are less widely distributed, yet some compounds from these groups, e.g., resveratrol and curcumin have received much attention for their multiple effects on various diseases [78]. We also would like to mention the terpenoids (isoprenoids), which are the largest class of natural products in plants and comprise more than 40,000 different structures. According to the number of isoprene molecules incorporated, they can be classified into hemiterpenes (C5), monoterpenes (C10), sesquiterpenes (C15), diterpenes (C20), triterpenes (C30), etc. Table 1 shows representatives for sesquiterpenes and diterpenes. We have also included a neolignan biphenol compound, i.e., honokiol. Despite the large number of structures only a few compounds have been identified as protein kinase inhibitors. Since most of the serine/threonine kinase inhibitors known so far belong to the flavonoids our focus in this review is on these compounds. 

Potentially, flavonoids, which are ATP mimetic, can target all 500 protein kinases. We have focused on a selection of serine/threonine protein kinases, which are part of the major signalling pathways as shown in Figure 1. The eleven kinases listed in Table 1 were identified in a linked analysis in PubMed inserting the natural compounds listed in Table 1: ((natural compound) AND protein kinase) AND cancer). Altogether 22 compounds from Table 1 were linked as described above. The numbers in Table 1 associated to the individual compounds indicate the number of hits. In the case of apigenin 188 publications were found when linked with the term protein kinase and cancer. The highest number under this linkage analysis that we found was 621 hits for curcumin. This does not come as a surprise since there is a plethora of research articles available on curcumin spanning already several decades. The number of hits increases on a daily basis, especially for the most popular compounds listed in row number one. We have foremost considered those kinases that belong to the signal transduction pathways shown in Figure 1.

The results shown in Table 2 come as no surprise. All of the listed kinases show a large agreement with respect to the natural compounds involved in the linked search. Although this approach may look trivial it demonstrates that the assumption of a general interaction of the kinases and natural compounds is as expected because of the binding to the ATP site. Although 9000 flavonoids have been so far identified, only a small selection has been investigated thoroughly with respect to protein kinase interaction. Hence, we postulate that many more kinase will show similar interaction patterns with natural compounds as seen in Table 2.

## 8. How Can One Cope with the Adverse Effects of Various Compounds Targeting Driver Gene Products? The Role of Small Molecule Inhibitors

A better understanding of signalling networks operating in cancer cells has helped to define the biology of malignant tumours and to demonstrate the importance of components of core signalling pathways that promote and sustain the tumour microenvironment. The observation that oncogenic mutations are responsible for the induction of molecular aberrations observed in cancer progression have boosted the development of small molecules inhibitors of oncogene products in order to target and weaken oncogenic signalling. Chemical compounds that target multiple critical protein kinases or specific ones (i.e., in the case of tumours that are highly dependent on a single genetic aberration) may turn out to be a clinical success, since the success rate for bringing cancer drugs to the market is very limited [6]. One of the major reasons is toxicity, which often can be observed in late stages of clinical development. A consideration pertinent to this is that the adverse effects that may derive from a particular compound can be considered as off-target toxicity. Driver protein kinase(s) play a crucial role in normal cells by controlling vital processes as cell growth and division, yet, when deregulated, they contribute to malignant transformation. Thus, inhibition of these kinases is a desirable option interfering with tumourigenesis. One could predict that the design of compounds able to specifically target aberrant protein kinase(s) and not their normal counterparts or to target protein kinases whose (over)expression is strictly required for cells undergoing the process of transformation would perhaps turn out to be a more effective strategy to treat cancer. In this respect, the survival of cancer cells depends on multiple factors which include not only the expression of oncogenic drivers but also proteins that do not per se cause cell transformation but are essential for tumour cell survival and possibly for coping with increased levels of various stressors. The concept of non-oncogene addiction [79] indeed reflects the notion that cancer cells heavily depend on this particular group of proteins whose expression is often found upregulated. Thus, the idea of harming their function and/or expression is considered an attractive treatment modality, as cancer cells would not tolerate these perturbations as easily as normal cells. We would like to focus on one protein kinase with respect to non-oncogene addiction.

### Protein Kinase CK2

Within the super family of protein kinases, protein kinase CK2 represents a convincing example of non-oncogene addiction [80]. CK2 is a highly conserved serine/threonine kinase composed of two catalytic subunits (i.e., α and/or α’) and two regulatory β-subunits with the ability to participate in the regulation of a broad range of cellular processes from motility to cell division, survival and metabolism [81,82,83]. Compelling evidence has shown that CK2 is involved in various stages of cell transformation and cancer development. Abnormally high levels of this enzyme have been consistently reported in all investigated cancer cell types but also in samples isolated from cancer patients while somatic mutations have hardly been detected [84]. CK2 activity and expression levels are elevated in many cancers of diverse genetic background including breast [85], lung [86], prostate [87], colorectal [88], renal [89,90] and leukaemias [91]. A more direct link between CK2 and cell transformation has been demonstrated in transgenic mice. Co-expression of CK2α and c-Myc results in polyclonal neonatal leukaemia [92]. Similarly, expression of Tal-1 transcription factor in transgenic mice leads to accelerated lymphoid malignancy in the presence of the CK2α transgene [93]. Xu et al. [94] showed a direct correlation between overexpression of CK2 and accelerated onset of lymphomas in mice heterozygous and homozygous for the p53 null allele, respectively, confirming the contribution of CK2 to tumourigenesis. This overwhelming collection of data strongly supporting the role of CK2 in cancerogenesis, has, so far, failed to provide a complete picture of the molecular mechanisms regulated by CK2 in the context of cell transformation and cancer. Nevertheless, the fact that tumour cells unlike normal cells rely on elevated levels of CK2 for sustaining the high proliferation pace and survival supports the notion that inhibition of this protein kinase may represent a clever way to treat most common types of malignant tumour by severely damage cancer cells while leaving healthy tissue largely unaffected. CK2 orchestrates a broad network of cellular functions and even if its expression has a modest effect on cell transformation, the control exerted on individual components of intracellular signalling cascades may altogether have a considerable impact on malignant transformation.

Numerous small molecules inhibitors of CK2 have been developed over the past 30 years displaying reduction in cancer cells viability and induction of cell death to a variable extent in tissue culture and animal models [95]. Two of the compounds most recently identified through high throughput screenings of natural compound libraries comprise 4-[(E)-(fluoren-9-ylidenehydrazinylidene)-methyl]benzoic acid (E9 [96,97]) and 1,3-Dichloro-6-[(E)-((4-methoxyphenyl)imino)methyl] diben- zo(b,d) furan-2,7-diol (D11 [98,99,100]). In particular, E9 was tested in vivo in murine xenograft models exhibiting significant antitumour effects without showing detrimental effects on mouse body weight and organ toxicity [97]. 

Overall, data obtained in vivo employing inhibitors of CK2, although preliminary, unequivocally demonstrate that inhibition of this enzyme results in anti-neoplastic effects in various types of cancer without causing life-threatening side effects providing evidence of a possible clinical benefit. 

## 9. Future Perspectives, Challenges and Limitations

Natural products are now considered as an alternative for synthetic drugs. These natural products are present in numerous sources like, plants, microorganisms, fungi, etc. Besides being non-toxic in nature they are considered less expensive. There are trends to show that the mainstream in pharmacological research is moving away from single molecule or single target approach to combinations and multiple target approaches [23]. In 2013, the FDA approved 1453 new chemical entities from which 40% comprised of natural products or analogues of natural compounds [101,102]. Natural products alone or in combination have been able to induce apoptosis as well as chemosensitised those cell lines that were resistant to conventional drugs. A key obstacle in the development of a specific inhibitor is the variation in efficacy observed in the cell line based experiments and rodent models during drug discovery phase leading to the final efficacy in patients [6]. Since the development of the first kinase inhibitor in the early 1980s, over 40 kinase inhibitors have received FDA approval for treatment of malignancies such a breast and lung cancer. Moreover, about 150 kinase-targeted drugs are in clinical phase trials, and many kinase-specific inhibitors are in the preclinical stage of drug development [6].

Kinase inhibitors symbolize a class of targeted cancer therapeutic agents with limited non-specific toxicities. Based on the latest update published in November 2018, forty-nine inhibitors with activity targeted to one or multiple kinases have been approved by FDA for clinical use of which the majority are directed against tyrosine kinases (http://www.brimr.org/PKI/PKIs.htm [6]). 

Only a few inhibitors directed against serine/threonine kinases, e.g., B-Raf are in clinical use [6]. Serine/threonine kinase inhibitors targeting mTOR, MAPK Aurora kinases and CK2 are under development for clinical application [103,104]. We have focused on the classical major signal transduction pathways shown in Figure 1. The focus is on natural compounds derived from plants, especially on flavonoids. Yet, it should not go unmentioned that many other protein kinases are in the limelight of current research in academia and in pharmaceutical companies. This concerns protein kinase B, polo-like kinase I, cyclin-dependent kinases and others. CDKs also have been targeted with natural compounds from other sources than plants, e.g., from marine organisms. This is a potential new source for finding further potent kinase inhibitors [105,106,107].

Plants are subject to many “attacks” by viruses, bacteria and fungi. Therefore, it was mandatory for plants to develop mechanisms of defence through so-called secondary metabolites, which usually are not as vital as primary metabolites such as carbohydrates and amino acids. An example of secondary metabolites are alkaloids, primarily composed of nitrogen, that are widely used in medicine. With currently more than 12,000 known structures, alkaloids represent one of the biggest groups of natural compounds. An exhaustive overview over the different classes of natural products is described in Springob et al., [108]. 

Most of the protein kinase inhibitors isolated from plants are flavonoids, which are part of the polyketides. But there are also other members of this chemical group such as the anthrones and anthraquinones with representatives such as emodin and hypericin. Both compounds have been shown to inhibit protein kinases such as protein tyrosine kinase p65lck and CK2 [109,110]. 

As with the alkaloids most of the members of the flavonoid group existed as valuable “folk medicine” beneficial for many other diseases than cancer. But even when shown to be useful for treating cancer, other mechanisms than interference with protein kinases have been described. 

Wine polyphenol extracts have been shown to arrest cell cycle, induce apoptosis through caspase activation, and induce changes in metalloproteinase (MMP) activities. Resveratrol, a stilbene found in many food sources and in red wine grapes is assumed to have multiple benefits on human health. It has been shown to inhibit protein kinases such as AKT, RAF, MEK/ERK1/2, CamKK and JNK (Table 2). Therefore it is tempting to speculate that it is the interference with the various protein kinases, which is responsible for the observed “anticancer effects”. Yet, this may be more apparent than real given the low amounts of resveratrol taken up by consumption of wine. Even the intake of the pure resveratrol compound would not be sufficient to have a measurable effect on cellular protein kinase activity. Moreover, given the fairly large number of different protein kinases that are affected by resveratrol, one cannot exclude negative side effects when large amounts are employed. The same argument applies to other natural compounds that have been described throughout this review.

At the moment there are too many different biological aspects known which are tempting to assume that they are responsible for the observed health effects. Each compound for itself is not efficient enough in plant extracts to account for a successful treatment of cancer. 

There are increasing reports of the isolation of protein kinase inhibitors from other sources, beside plants. Recently protein kinase inhibitors have been isolated from marine sources [106,107]. This is a new and promising approach in the search for new types of kinase inhibitors. However, since there are so many other factors: environmental, genetic and personal habits of nutrition, physical exercise, etc.; food-related arguments alone are not enough. 

The search for the ‘magic bullet’, where a particular molecule is targeted may give way for a broader application of flavonoids, simply through the fact, that they intervene on many cellular ‘battle fields’. Perhaps this fact will lead in the future to a successful anticancer therapy when applied correspondingly.

## Figures and Tables

**Table 1:** Classification of flavonoids and other polyphenolic compounds.

Flavones	Flavonols	Flavanones	Flavanonols	Isoflavones	Flavan-3-ols/Catechins	Anthocyanins	Sesquiterpenes	Diterpenes	Neolignan Biphenols	Coumestans	Alkaloids
Apigenin 188	Quercetin 344	Naringenin 37	Silibinin 115	Genistein 794	Epigallocatechin-3-gallate 309	Delphinidin 33	Parthenolide 52	Oridonin 50	Honokio l39	Wedelolactone 14	Curcumin 621
Luteolin 116	Kaemphero l86	Hesperidin 24	Silymarin 130	Daidzein 68		Cyanidin 24					
Baicalein 67	Fisetin 47	Hesperetin 18	Taxifolin 6								

The numbers indicate citations found for the compounds using a linked search: *((compound) AND protein kinases) AND* cancer) in PubMed.

**Table 2:** Selection of serine/threonine-specific kinases (STKs) shown to be inhibited by natural compounds.

Protein Kinase	Natural Compound
PI3K	Apigenin, Fisetin, Naringenin, Silibinin, Parthenolide, Oridonin, Honokiol, Genistein, EGCG, Taxifolin, Ellagic acid, Emodin, Curcumin
AKT	Apigenin, Fisetin, Quercetin, Naringenin, Silibinin, Parthenolide, Oridonin, Curcumin EGCG, Luteolin, Resveratrol, Genistein, Taxifolin, Wedelolactone, Ellagic acid, Emodin, Harmine, Curcumin
mTOR	Apigenin, Quercetin, Genistein, EGCG, Curcumin, Oridonin, Silibinin, Wedelolactone, Curcumin
GSK3β	Apigenin, Curcumin, Berberine, Resveratrol, Curcumin, Luteolin, Quercetin, Curcumin
CK2	Apigenin, Coumestrol, Resorufin, Gallaflavin, Fisetin, Nortangeretin, Ellagic acid
RAF	Curcumin, EGCG, Resveratrol, Parthenolide
MEK/ERK1/2	Apigenin, Quercetin, Silibinin, Oridonin, Genistein, Parthenolide, Genistein, Honokiol, Cyanidin, Berberine, Quercetin, Ellagic acid, Emodin
PKC	Apigenin, Wedelolactone, Curcumin
CamKK	Apigenin, Curcumin, Resveratrol, Berberine, EGCG (stimulation)
IKK	Apigenin, Wedelolactone
JNK	Apigenin, Quercetin, Silibinin, Cyanidin, Parthenolide, Hesperetin
